# Are Sensitivity to Punishment, Sensitivity to Reward and Effortful Control Transdiagnostic Mechanisms Underlying the Eating Disorder/Obesity Spectrum?

**DOI:** 10.3390/nu13103327

**Published:** 2021-09-23

**Authors:** Laurence Claes, Glenn Kiekens, Els Boekaerts, Lies Depestele, Eva Dierckx, Sylvia Gijbels, Katrien Schoevaerts, Koen Luyckx

**Affiliations:** 1 Faculty of Psychology and Educational Sciences, KU Leuven, 3000 Leuven, Belgium; glenn.kiekens@kuleuven.be (G.K.); koen.luyckx@kuleuven.be (K.L.); 2 Faculty of Medicine and Health Sciences, University Antwerp, 2000 Antwerp, Belgium; 3 Center for Contextual Psychiatry, Department of Neurosciences, KU Leuven, 3000 Leuven, Belgium; 4 Obesity Centre Hasselt, Jessa Hospital, 3500 Hasselt, Belgium; els.boekaerts@jessazh.be (E.B.); sylvia.gijbels@jessazh.be (S.G.); 5 Psychiatric Hospital Alexianen Zorggroep Tienen, 3300 Tienen, Belgium; lies.depestele@azt.broedersvanliefde.be (L.D.); Eva.Dierckx@vub.be (E.D.); katrien.schoevaerts@azt.broedersvanliefde.be (K.S.); 6 Department of Clinical Psychology, Vrije Universiteit Brussel, 1050 Brussels, Belgium; 7 UNIBS, University of the Free State, Bloemfontein 9300, South Africa

**Keywords:** sensitivity to reward, sensitivity to punishment, effortful control, eating disorders, obesity, bariatric surgery

## Abstract

Although it has been postulated that eating disorders (EDs) and obesity form part of a broad spectrum of eating- and weight-related disorders, this has not yet been tested empirically. In the present study, we investigated interindividual differences in sensitivity to punishment, sensitivity to reward, and effortful control along the ED/obesity spectrum in women. We used data on 286 patients with eating disorders (44.6% AN-R, 24.12% AN-BP, and 31.82% BN), 126 healthy controls, and 640 Class II/III obese bariatric patients (32.81% Class II and 67.19% Class III) with and without binge eating. Participants completed the behavioral inhibition and behavioral activation scales, as well as the effortful control scale, to assess sensitivity to punishment and reward and effortful control. Results showed that patients with EDs scored significantly higher on punishment sensitivity (anxiety) compared to healthy controls and Class II/III obese patients; the different groups did not differ significantly on reward sensitivity. Patients with binge eating or compensatory behaviors scored significantly lower on effortful control than patients without binge eating. Differences in temperamental profiles along the ED/obesity spectrum appear continuous and gradual rather than categorical. This implies that it may be meaningful to include emotion regulation and impulse regulation training in the treatment of both EDs and obesity.

## 1. Introduction

Several authors postulate that eating disorders (EDs) and obesity form part of a broad spectrum of eating- and weight-related disorders [1]. Concerning weight status, anorexia nervosa of the restrictive type (AN-R) and the binge eating/purging type (AN-BP) are characterized by being underweight (BMI < 18.5 kg/m^2^), and bulimia nervosa (BN) by normal weight (BMI 18.5–24.9 kg/m^2^). Within obesity (BMI ≥ 30 kg/m^2^), researchers differentiate between Class I (BMI 30–34.9 kg/m^2^), Class II (BMI 35–39.9 kg/m^2^), and Class III obesity (BMI ≥ 40 kg/m^2^) [2]. With respect to eating-related behaviors, patients with AN-R mainly engage in severe food restriction, whereas patients with AN-BP also report binge eating and purging behaviors (e.g., vomiting, laxative abuse, etc.) besides food restriction. Patients with BN and binge eating disorder (BED) are characterized by regular episodes of binge eating with and without compensatory behaviors, respectively [3]. The causes of obesity are diverse, including genetic, environmental, and behavioral aspects of excessive energy intake, partitioning, and expenditure [4]. Obese patients with a comorbid eating disorder (mainly BED, which is reported in 30% of obese patients) report more eating and weight-related pathology, as well as more general and personality psychopathology [1,5,6], compared to obese patients without BED.

The question remains open as to why patients with AN are able to highly restrict their food intake and become emaciated, whereas other patients binge and overconsume with and without purging behaviors [7,8]. Part of the explanation might be found in interindividual differences in reactive (bottom-up) and regulative (top-down) temperament [7,8]. According to dual-process models, ED behaviors result from the interplay of bottom-up processes (e.g., sensitivity to punishment and sensitivity to reward) and top-down processes (e.g., effortful control) [9]. One of the most applied models of reactive temperament that can be used to explain individual variations in food intake is reinforcement sensitivity theory (RST) [7,10,11], which encompasses two primary motivational systems: the behavioral inhibition system (BIS) and the behavioral activation system (BAS). The BIS is sensitive to stimuli that signal conditioned punishment and the omission/termination of reward and is involved in behavioral inhibition [12]. The BIS is related to personality traits, such as the Big Five neuroticism dimension and Cloninger’s harm avoidance dimension [13,14]. The BAS is sensitive to stimuli that signal unconditioned reward and relief from punishment and is involved in approach behavior [12]. The BAS is related to personality traits, such as extraversion and novelty seeking [13,14]. Over the years, the RST has included a third system: the fight–flight system [15]. In 2000, Gray and McNaughton [16] presented a revised version of the RST in which the BAS is responsive to (un)conditioned stimuli of reward and the fight–flight–freeze system is responsive to (un)conditioned stimuli of punishment, while the BIS resolves goal conflicts (e.g., approach-avoidance conflicts).

Besides reactive temperament (bottom-up, automatic), regulative (top-down, controlled) temperament or executive control [17] can also play an important role in the regulation of food intake and weight. Self-regulation is often used interchangeably with terms such as effortful control [18] and self-regulation [19]. Effortful control is also related to particular personality traits, such as the Big Five conscientiousness dimension and Cloninger’s self-directedness dimension [17]. It is assumed that effortful control can directly influence ED behaviors or moderate the association between reactive temperamental traits and ED psychopathology, in the sense that a high level of effortful control might help individuals control their reactive temperament and decrease their risk of developing ED psychopathology [20].

Up until now, most studies have investigated BISBAS reactivity and effortful control in patients with EDs or obesity with and without BED (often compared to healthy controls) separately; but none of these studies included patients situated over the whole spectrum of ED/Obesity within a single study. In what follows, we present an overview of the literature on the sensitivity of reward/punishment and effortful control in patients with EDs and obesity.

### 1.1. Punishment Sensitivity

The most applied measures to assess sensitivity to punishment (BIS) and reward (BAS) are the BISBAS scales [21] and the sensitivity to punishment and sensitivity to reward questionnaire (SPSRQ) [22], both based on the original RST theory. The BISBAS scales focus on the general disposition towards reward (e.g., “When I’m doing well, I love to keep at it.”) and punishment, whereas the SPSRQ items often include specific situational triggers related to punishment and reward (e.g., “Do you often meet people that you find physically attractive?”). Patients with EDs (AN-R, AN-BP, or BN) typically report significantly higher scores on punishment sensitivity (BIS/SP) compared to healthy controls [8,23,24,25,26,27,28,29]. When comparing different ED subtypes, most studies did not find significant differences among AN-R, AN-BP, or BN patients [8,25,29,30]. Two studies [23,27] showed that AN-R patients scored significantly higher on sensitivity to punishment than AN-BP or ED-PB (AN-BP + BN) patients, whereas other studies found the opposite [24] or no significant differences [31].

Studies comparing patients with obesity to healthy controls on punishment sensitivity are rather scarce. Class I obese patients without BED scored significantly lower on punishment sensitivity compared to healthy controls [32], whereas Class I obese patients with BED did not significantly differ from healthy controls [33]. Class II obese patients with and without BED scored significantly higher on punishment sensitivity than healthy controls [32,34]. No study has compared patients with Class III obesity with and without BED to healthy controls with respect to punishment sensitivity. When comparing obese patients (Class II, III) with and without BED on punishment sensitivity, patients with and without BED did not differ from each other [6,34]; no studies were performed in Class I obese patients with and without BED.

Higher punishment sensitivity in patients with EDs, as compared to healthy controls, seems to be linked to their symptomatology. Patients with AN-R relate their self-worth to their weight [35] and are afraid of gaining weight; their strict dieting can be considered as a way to avoid anxiety for weight gain [7,8]. Several studies have demonstrated a positive association between sensitivity to punishment and restrained eating in both adolescents and young adults [20,36]. Patients with binge eating (AN-BP, BN) and obesity, on the contrary, often use food to comfort or soothe themselves and to escape from negative feelings [37]. Davis [38], for example, showed a positive association between sensitivity to punishment and the symptoms of binge eating. The compensatory behaviors of patients with AN-BP/BN can again be considered as a way to avoid weight gain [7,8].

### 1.2. Reward Sensitivity

When comparing ED patients to healthy controls on reward sensitivity, we need to differentiate between the studies that use the SPSRQ and those that use the BISBAS scales to assess reward sensitivity. As mentioned before, the SPSRQ assesses specific types of rewarding situations (i.e., physical attractiveness, or social approval), whereas the BISBAS scales assess more general reward sensitivity [24]. Studies using the BAS scale to assess reward sensitivity [8,23] have shown that AN-R patients score significantly lower on reward sensitivity compared to healthy controls, whereas AN-BP and BN do not differ significantly from healthy controls. When studies combine AN-R and AN-BP in one group, the difference between them and the healthy controls disappears. Studies using the SR scale show that AN-R, AN-BP, and BN patients score significantly higher on reward sensitivity compared to healthy controls [8,24,26]. However, Glashouwer et al. [24] showed that the differences between EDs and healthy controls disappeared when items that assessed appearance/social reward were removed from the SR scale. When comparing different subtypes of EDs on the BAS scale, most studies did not find significant differences between AN-R and AN-BP [26,27,31] or AN-R, AN-BP, and BN [23,29]. Studies that found significant differences between ED subtypes showed that binge eating/purging patients (AN-BP, BN) scored significantly higher on BAS fun seeking compared to restrictive AN patients [8,23,30]. Comparing ED subtypes utilizing the SR scale showed that patients with AN-R and AN-BP did not differ significantly from each other on the SR scale, whereas BN patients scored significantly higher compared to AN-R patients.

When comparing patients with obesity to healthy controls on reward sensitivity, patients with Class I obesity without BED did not differ from healthy controls on reward sensitivity [32], whereas patients with Class I obesity with BED scored significantly higher on sensitivity to reward when compared to healthy controls [33]. Patients with Class II obesity with and without BED also scored significantly higher on reward sensitivity (both BAS/SR scales) as compared to healthy controls [34]. No study compared patients with Class III obesity with and without BED to healthy controls on sensitivity to reward.

When comparing obese patients, obese patients (Class II/III) with and without BED did not differ significantly from each other on reward sensitivity [6,34]; no studies were performed in Class I obese patients. The higher reward sensitivity in moderate/extreme obese patients makes them possibly more vulnerable to rewarding (fatty/sugary) food in our obesogenic society, which may partially explain the overconsumption of food and their subsequently becoming overweight [7,39]. Several studies have shown positive associations between sensitivity to reward and emotional overeating, preference for high fat food, binge eating, and food cravings [39].

### 1.3. Effortful Control

Effortful control and its subdimensions (inhibitory, activation, and attentional control) can be measured using the effortful control scale (ECS) from the adult temperament questionnaire short form (ATQ-SF) [40,41]. The perception of low control and the desire for higher control in AN patients (and in particular, patients with AN-R) are psychological variables that were frequently considered in the papers of the original ED theorists, including Bruch [42], Crisp [43], Garfinkel and Garner [44], and Selvini Palazzoli [45]. In the 1980s, Eric Button [46], described AN as a quest for control. Indeed, studies comparing ED subtypes utilizing the ECS show that AN-R patients score significantly higher on effortful control than AN-BP/BN patients, particularly on the subscales for inhibitory and activation control [23,31]. Studies comparing obesity subtypes utilizing Cloninger’s self-directedness or Rothbart’s effortful control scales do not show differences among Class I, II, and III obesity categories with respect to self-directedness; however, obese patients with BED score significantly lower on self-directedness/effortful control, compared to obese patients without BED [6,47]. Thus, it seems that a lack of effortful control may make ED/obese patients vulnerable to binge eating (and purging).

### 1.4. The Present Study

The aim of the present study is to investigate temperamental differences on BIS/BAS and effortful control along the ED/obesity spectrum. We collected data from female AN-R, AN-BP, and BN patients, healthy controls, and Class II and Class III obese bariatric patients with and without binge eating within the age range 18–65. Thus, we add to the existing literature by comparing ED, healthy controls, and obesity patients in one study on both executive/top-down and reactivity measures of temperament.

With respect to punishment sensitivity (BIS), we expect that ED and obese patients will score significantly higher compared to healthy controls. Within the ED/obesity subtypes, we do not expect significant differences on punishment sensitivity [48,49].

With respect to reward sensitivity (BAS), we expect no significant differences between ED and healthy controls, with the exception of a potentially lower reward reactivity in AN-R patients compared to healthy controls [48,49]. However, we do expect higher levels of reward sensitivity in obese patients compared to healthy controls. Within obesity subtypes with and without BED, we again do not anticipate differences in BAS reactivity [6,47].

Finally, concerning top-down control, we expect that patients with ED/obesity will score significantly lower than healthy controls [6,23,31,47], whereas patients with binge eating and/or purging (AN-BP, BN, obesity Class II/III with binge eating), are hypothesized to score lower on effortful control compared to patients without binge eating and/or purging (AN-R, obesity Class II/III without binge eating) [6,23,31].

## 2. Materials and Methods

### 2.1. Participants & Procedure

Female patients with EDs, healthy controls, and individuals with obesity (from ages 18 to 65) were sampled out of three different data collections.

The sample of ED inpatients consisted of 286 patients, of whom 126 (44.06%) were diagnosed as AN-R, 69 (24.12%) as AN-BP, and 91 (31.82%) as BN through a clinical interview, and cross-validated by the eating disorder evaluation scale (EDES) [50]. The mean age and BMI of all ED subtypes are displayed in Table 1. The three ED subtypes did not differ significantly in age and were significantly younger than healthy controls and patients with obesity. Concerning BMI, AN patients (AN-R/AN-BP) had a significantly lower BMI than BN patients and healthy controls (normal weight) and Class II/III obese patients (severe overweight). All data were collected at the admission of these patients at a specialized inpatient treatment unit for EDs in Flanders, the Dutch-speaking part of Belgium.

The sample of female patients with Class II/III obesity was collected during an intake for a bariatric surgery trajectory at a general hospital in the Dutch-speaking part of Belgium. The presence or absence of binge eating was determined by two items (overeating + loss of control) of the Dutch version of the eating disorder examination questionnaire (EDEQ; see Section 2.2. Instruments). About 210 patients were diagnosed with Class II obesity, of whom 72 (34.29%) engaged in binge eating, and 430 patients with Class III obesity, of whom 141 (32.79%) engaged in binge eating. These comorbidity rates of binge eating are in line with prior research [6]. The mean age and BMI of the individuals with Class II/III obesity with and without binge eating are displayed in Table 1. All patients with obesity were significantly older than patients with ED, and were similar in age to healthy controls (except patients of Class II obesity without BED, who were significantly older). Within the obesity subtypes (Class II and Class III), patients with binge eating were significantly younger than those without binge eating. Concerning BMI, obese patients had significantly higher BMIs compared to patients with AN (underweight), and BN and healthy controls (normal weight). Obesity Class III patients also had a significantly higher BMI than patients with Class II obesity.

Finally, the 126 healthy controls were collected from the Flemish-speaking general population of Belgium, taking into account the distribution of the Flemish population’s age, gender, and education. The mean age and BMI of the healthy controls are shown in Table 1. Healthy controls were significantly older than patients with an ED, and similar in age to patients with obesity (except patients of Class II obesity without binge eating, who were older). Concerning BMI, healthy controls had a normal BMI, as did BN patients. However, their BMI was higher than patients with AN, and lower than patients with Class II/III obesity.

All patients and healthy controls gave their informed consent to use their data anonymously for research purposes. The three data collections from which the data were pooled were approved by the ethical committee of the Faculty of Psychology and Educational Sciences (healthy controls) and/or the medical ethical committee of the medical institute in which the patients (with Eds and/or obesity) were treated. Given that the groups did significantly differ regarding age, this variable was included as a confounding variable in all analyses. Although groups also differed with respect to BMI, we did not include BMI as control variable, given that it is an essential characteristic of the diagnostic groups.

### 2.2. Instruments

To determine the BMI of the participants, we calculated their weight in kilograms and divided this by their height in m^2^. To determine the presence or absence of “binge eating” (not BED), we asked two questions from the EDEQ [51]: “Over the past 28 days, how many times have you eaten what other people would regards as an unusually large amount of food (given the circumstances)?”; and “On how many of these times did you have a sense of having lost control over your eating (at the time you were eating)?”. Patients who reported eating unusually large amounts of food while losing control over their eating were considered to engage in binge eating.

Sensitivity to reward and punishment was measured utilizing the behavioral inhibition/behavioral activation system (BISBAS) scales [21]. The BISBAS scales consist of 20 4-point Likert scale items, ranging from 1 “I strongly agree” to 4 “I strongly disagree”. The BIS scale assesses worry concerning potential punishment in the future (*n* = 7, α = 0.80 in the present study, e.g., ‘‘I worry about making mistakes.’’), and the BAS scale assesses sensitivity to reward (*n* = 13, α = 0.81). The BAS scale (disinhibition) exists of three subscales, measuring BAS drive (*n* = 4, α = 0.74, e.g., ‘‘When I want something, I usually go all-out to get it”), BAS fun seeking (*n* = 5, α = 0.52, e.g., ‘‘I often act on the spur of the moment’’), and BAS reward responsiveness (*n* = 5, α = 0.61, e.g., ‘‘When I’m doing well at something, I love to keep at it’’).

Effortful/executive control was measured using the effortful control scale (ECS) from the adult temperament questionnaire short form (ATQ-SF) [40,41]. The ECS exists of 19 7-point Likert scale items, ranging from 1 “not at all applicable” to 7 “completely applicable” (α = 0.80 in the present study). The ECS has three subscales: attention control is the capacity to focus and shift attention when necessary (*n* = 5, α = 0.73, e.g., ‘‘It is very hard for me to focus my attention when I am distressed.’’(reversed)); inhibitory control is the capacity to suppress inappropriate approach behavior (*n* = 7, α = 0.54, e.g., ‘‘I can easily resist talking out of turn, even when I’m excited and want to express an idea.’’); and finally, activation control refers to the capacity to act when there is a strong tendency to avoid it (*n* = 7, α = 0.68, e.g., ‘‘If I think of something that needs to be done, I usually get right to work on it.’’).

### 2.3. Analyses

Descriptive statistics are reported as means with associated errors. To compare the different ED/HC/obesity groups on the basis of their sensitivity to punishment, sensitivity to reward, and effortful control, we performed ANCOVAs with the group as the independent variable, temperamental dimensions as dependent variables, and age as a covariate. When the ANCOVAs showed significant results, simple contrasts were used to evaluate subgroup differences.

## 3. Results

The means (standard errors) of the different temperament measures for each ED/HC/obesity group are displayed in Table 1.

### 3.1. Punishment Sensitivity

**Figure 1:**
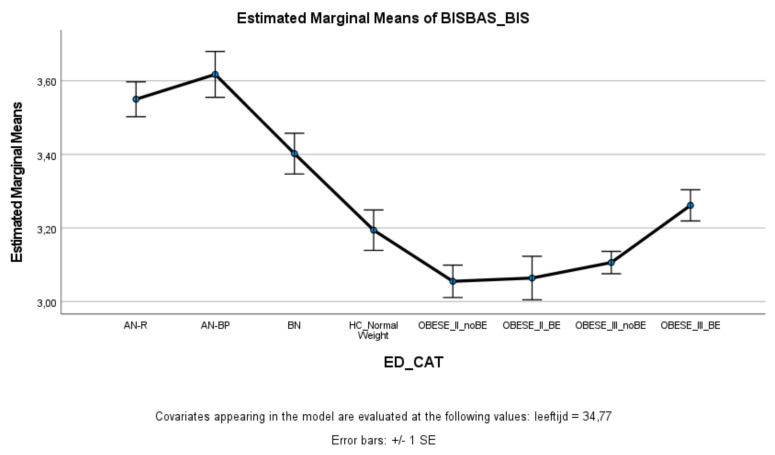
Means (standard errors) of the behavioral inhibition scale scores for eating disorders, healthy controls, and obesity groups, controlled for age.

With respect to punishment sensitivity (Figure 1), all ED groups scored significantly higher on punishment sensitivity (BIS) compared to healthy controls and patients with obesity. Patients with AN (AN-R+AN-BP) scored significantly higher on punishment sensitivity than patients with BN. Obesity groups did not differ significantly from healthy controls (except Class II obesity—BE) on punishment sensitivity, and scored significantly lower on punishment sensitivity than ED groups. Within the obesity groups, Class III obesity patients with binge eating reported significantly higher punishment sensitivity levels than the other obesity groups.

### 3.2. Reward Sensitivity

**Figure 2:**
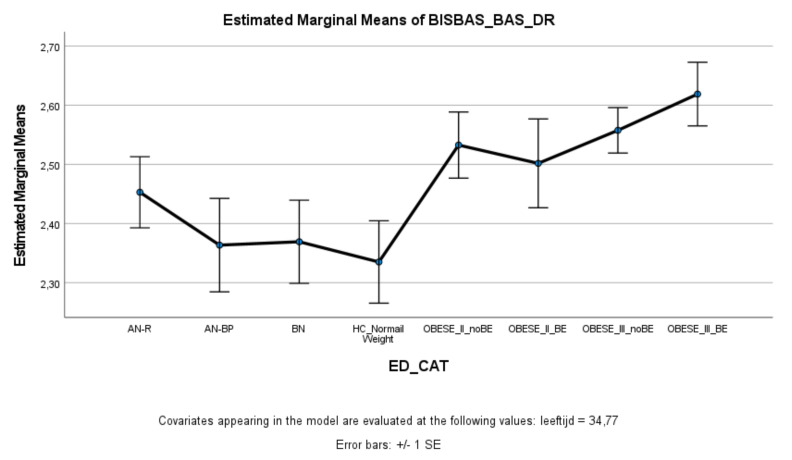
Means (standard errors) of the behavioral activation scale—drive scores for eating disorders, healthy controls, and obesity groups, controlled for age.

With respect to the BAS total score and the fun seeking and reward responsiveness subscales, the ED, healthy controls, and obesity groups did not differ significantly from each other. However, on the BAS drive subscale (Figure 2), patients with Class III obesity with and without binge eating scored significantly higher on drive compared to patients with AN-BP, BN, and healthy controls. Patients with Class III obesity and binge eating also scored higher on the BAS drive subscale than patients with AN-R. There were no significant differences between patients with Class II and Class III obesity.

### 3.3. Effortful Control

**Figure 3:**
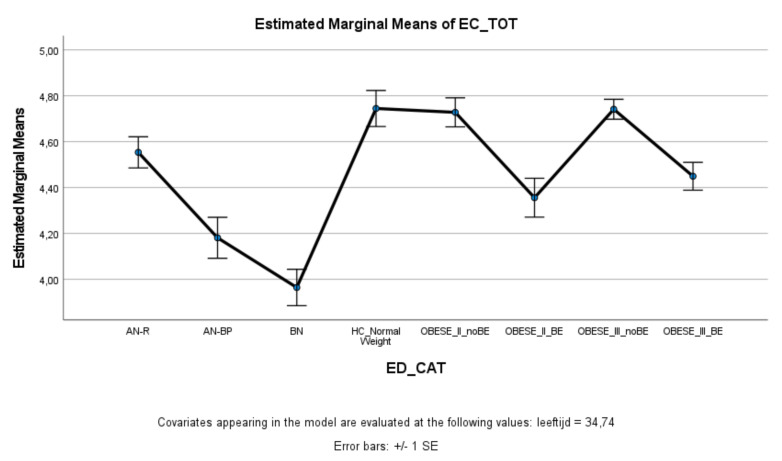
Means (standard errors) of the effortful control scale—total control scores for eating disorders, healthy controls, and obesity groups, controlled for age.

**Figure 4:**
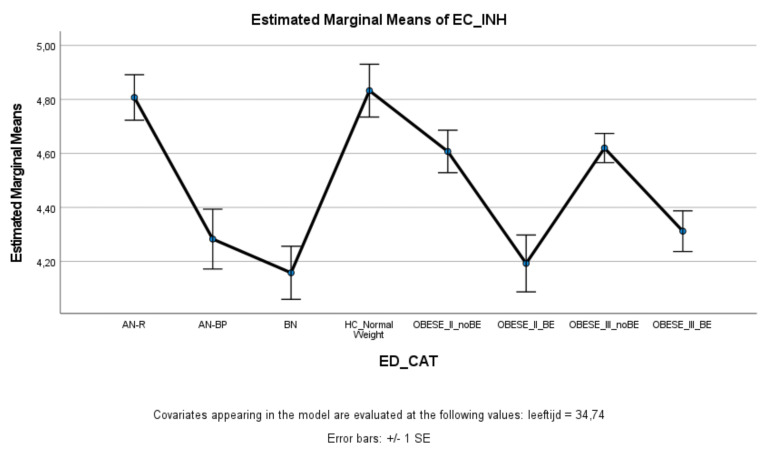
Means (standard errors) of the effortful control scale—inhibitory control scores for eating disorders, healthy controls, and obesity groups, controlled for age.

**Figure 5:**
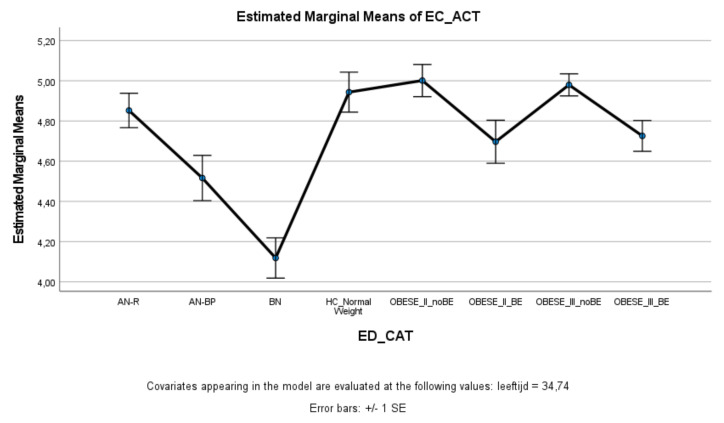
Means (standard errors) of the effortful control scale—activation control scores for eating disorders, healthy controls, and obesity groups, controlled for age.

**Figure 6:**
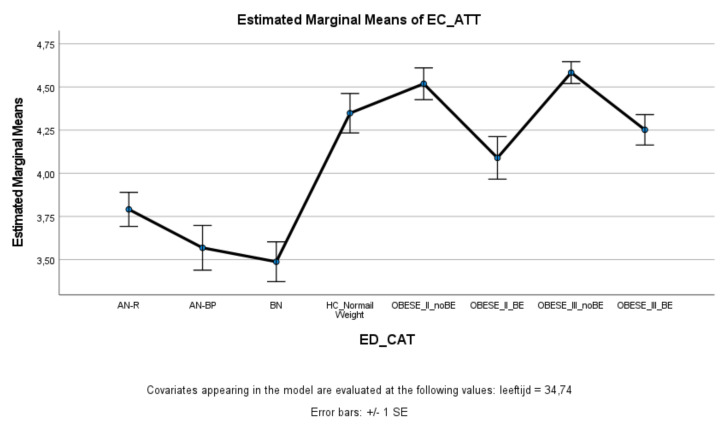
Means (standard errors) of the effortful control scale—attentional control scores for eating disorders, healthy controls, and obesity groups, controlled for age.

ED and obese groups with binge eating (AN-BP, BN, Class II obesity + BE, Class III obesity + BE) scored significantly lower on effortful control (total score and inhibitory control) compared to healthy controls, whereas ED and obese groups without binge eating (AN-R, Class II obesity—BE, Class III obesity—BE) did not significantly differ from healthy controls (Figure 3 and Figure 4). Within the ED groups, patients with AN-BP/BN scored significantly lower on effortful control (total, inhibitory, and activation) compared to patients with AN-R (Figure 3, Figure 4 and Figure 5), whereas in the obesity groups, obese patients with binge eating scored significantly lower on all measures of effortful control compared to obese patients without binge eating. Obese patients without binge eating scored similarly to healthy controls on activation and attentional control (Figure 5 and Figure 6).

## 4. Discussion

The present study investigated sensitivity to punishment, sensitivity to reward, and effortful control along the ED/obesity spectrum in women. Based on the findings, we can conclude that ED groups are significantly more sensitive to punishment than healthy controls and obese groups. Concerning reward sensitivity, patients with morbid Class III obesity scored significantly higher on BAS drive compared to ED groups (except AN-R) and healthy controls. Finally, all ED/obese groups with binge eating reported significantly lower levels of effortful control (particularly inhibitory control) compared to healthy controls and patients without binge eating.

### 4.1. Sensitivity to Punishment

The different ED/obese groups’ temperamental profiles can help us understand their disturbed eating patterns. The fact that all ED patients are more sensitive to punishment confirms previous studies’ findings [25] and can explain the high comorbidity between EDs and anxiety disorders [52]. The high scores on punishment sensitivity can also explain why people with an ED are afraid of weight gain and can resist the temptation to food (as a way to avoid this weight gain) [7,53].

### 4.2. Effortful Control

Variability in effortful control can also help explain differences between restrictive and binge eating/purging ED subtypes. While restrictive ED patients show similar levels of effortful control as healthy controls (except for attentional control), patients with binge eating/purging behaviors (AN-BP, BN) score significantly lower on effortful control. The lack of effortful control can explain why these patients lose control over their eating behaviors and eat comfort (sugary/fatty) food to deal with their negative effects [37], and then engage in compensatory purging behaviors to avoid weight gain [7,53]. Within this subgroup of obese patients, the lack of effortful control is clearly related to the presence of binge eating behaviors as well. Several studies have shown that obese patients with binge eating tend to have more comorbidity with impulsive psychopathology [54,55] than obese patients without binge eating. Other studies investigating subtypes of obese patients have also found support for the notion of a more resilient subgroup of obese patients and an emotional/behavioral dysregulated group, characterized by low levels of effortful control, psychological complaints, and avoidant and depressive coping patterns [23].

### 4.3. Reward Sensitivity

Finally, concerning reward sensitivity, we did not find significant differences between the ED, obese, and healthy control groups for BAS reactivity. While we did anticipate a potential difference between AN-R and healthy controls, and between obese patients and healthy controls, some studies also did not find differences when using the BISBAS scales [1,49]. In the present study, only patients with Class II obesity without binge eating and Class III obesity with and without binge eating scored significantly higher on BAS drive than ED and healthy controls. This finding possibly refers to the high drive of obese patients to attain their goal, i.e., the bariatric surgery.

### 4.4. Clinical Implications

The present study has several clinical implications. Given the high levels of punishment sensitivity, it is essential that the treatment of patients with ED focuses on acquiring emotion regulation skills to help patients cope with emotional distress [37]. This is not surprising, given that EDs are often considered anxiety disorders. Evidence-based treatments of EDs, such as CBT-E and DBT-E [56,57,58,59], often include strategies to deal with emotions and to replace maladaptive ED behaviors with more adaptive emotion regulation strategies [37]. Furthermore, for binge eating and/or purging patients, the lack of effortful control certainly needs attention. First of all, it is important that patients focus on the aims of the treatment (which can be complicated by difficulties in attentional control) and learn to steer their behaviors (which can be complicated by difficulties in inhibitory/activation control). Furthermore, we know from prior research that a lack of effortful control/self-directedness can increase drop-out rates and worsen outcomes in patients with ED and obesity [60]. Therefore, impulse regulation strategies are included in evidence treatments for ED and obesity (e.g., CBT) [61]. Dalle Grave et al. [60], for example, conclude that CBT techniques to increase self-control, such as “setting short-term and achievable goals, developing adaptive coping behaviors through problem-solving in order to achieve these goals, and developing the confidence that they possess the resources required to achieve these goals (via progressive increases in self-efficacy through mastery experiences)”, could decrease drop-out rates and improve therapy outcomes for patients with obesity [60], (p. 35). Adapting the evidence-based treatments to the temperamental profile of our patients will probably also decrease drop-out rates and further improve our treatments [62,63].

### 4.5. Limitations and Suggestions for Future Research

Besides the strengths of our study, several limitations need to be discussed and addressed for future studies. First of all, our sample solely consists of female patients with ED/obesity, and healthy controls. Furthermore, we lack a group of outpatients with an ED, ‘overweight’ people, a group with Class I obesity ± BE, and a group of Class II/III obese patients ± BE who did not apply for bariatric surgery. Therefore, future studies certainly need to include male ED in/outpatients, HC, and overweight people, as well as Class I obese and Class II/III obese ± BE who do and do not seek bariatric surgery. Secondly, binge eating was assessed through two items of the EDE-Q. In future studies, it would be better to use the diagnostic criteria of the DSM-5 binge eating disorder to assess the presence or absence of a BED. Thirdly, all temperament dimensions are assessed by means of self-report questionnaires, which can lead to problems of social desirability, while a few subscales also have rather small internal consistency coefficients (e.g., the BAS fun seeking scale). Therefore, future studies could include interviews or performance-based measures to assess sensitivity to punishment/reward and effortful control [17]. Notwithstanding these limitations, this study is one of few studies which addressed reward and punishment sensitivity and (lack of) effortful control along the weight/eating disordered spectrum in women. Hence, the main finding of our study is that temperamental differences on these dimensions are continuous and gradual, which implies that clinicians should incorporate emotion regulation and impulse regulation training transdiagnostically when working with patients suffering from ED and obesity.

## Figures and Tables

**Table 1:** Means (standard deviations/errors) of Age, BMI, the Behavioral Inhibition and Behavioral Activation Scales and the Effortful Control Scale and subscales for eating disorders, healthy controls and Class II/III obese bariatric groups controlled for age.

	AN-R (*N* = 12)		AN-BP (*N* = 69)		BN (*N* = 89)		HC (*N* = 84)		Class II Obesity − BE (*N* = 138)		Class II Obesity + BE (*N* = 72)		Class III Obesity − BE (*N* = 289)		Class III Obesity + BE (*N* = 140)		*F*
	M	(SD)	M	(SD)	M	(SD)	M	(SD)	M	(SD)	M	(SD)	M	(SD)	M	(SD)	
Age	24.60 _a_	(6.17)	24.52 _b_	(5.04)	24.41 _c_	(5.22)	38.05 _a,b,c,d_	(12.15)	42.07 _a,b,c,d,e_	(10.19)	37.19 _a,b,c,e,f_	(11.07)	39.42 _a,b,c,e,g_	(12.74)	35.53 _a,b,c,e,g_	(11.75)	58.55 ***
BMI	15.40 _a_	(1.66)	16.27 _b_	(1.67)	20.96 _a,b,c_	(1.77)	21.65 _a,b,d_	(1.74)	37.52 _a,b,c,d,e_	(1.45)	37.78 _a,b,c,d,f_	(1.58)	43.74 _a,b,c,d,e,f,g_	(4.03)	43.89 _a,b,c,d,e,f_	(4.32)	2223.92 ***
	M	(SE)	M	(SE)	M	(SE)	M	(SE)	M	(SE)	M	(SE)	M	(SE)	M	(SE)	
BIS-TOT	3.55 _a_	(0.05)	3.62 _b_	(0.06)	3.40 _a,b,c_	(0.06)	3.19 _a,b,c,d_	(0.06)	3.06 _a,b,c,d,e_	(0.04)	3.06 _a,b,c,f_	(0.06)	3.11 _a,b,c,g_	(0.03)	3.26 _a,b,c,e,f,g_	(0.04)	15.33 ***
BAS-TOT	2.79	(0.04)	2.74	(0.06)	2.78	(0.05)	2.75	(0.05)	2.79	(0.04)	2.85	(0.05)	2.85	(0.03)	2.92	(0.04)	1.91
BAS-DR	2.45 _a_	(0.06)	2.36 _b_	(0.08)	2.37_c_	(0.07)	2.34 _d_	(0.07)	2.53 _d,e_	(0.06)	2.50 _f_	(0.08)	2.56 _b,c,d,g_	(0.04)	2.62 _a,b,c,d_	(0.05)	2.70 **
BAS-FS	2.60	(0.05)	2.67	(0.07)	2.71	(0.06)	2.65	(0.06)	2.60	(0.05)	2.74	(0.06)	2.64	(0.03)	2.76	(0.05)	1.61
BAS-RR	3.20	(0.04)	3.10	(0.06)	3.16	(0.05)	3.17	(0.05)	3.16	(0.04)	3.23	(0.06)	3.24	(0.03)	3.29	(0.04)	1.80
EC-TOT	4.55 _a_	(0.07)	4.18 _a,b_	(0.09)	3.96 _a,c_	(0.08)	4.75 _b,c,d_	(0.08)	4.73 _b,c,e_	(0.06)	4.36 _c,d,e,f_	(0.09)	4.74 _a,b,c,f,g_	(0.04)	4.45 _b,c,d,e,g_	(0.06)	14.45 ***
EC-INH	4.81 _a_	(0.08)	4.28 _a,b_	(0.11)	4.16 _a,c_	(0.10)	4.83 _b,c,d_	(0.10)	4.61 _b,c,e_	(0.08)	4.19 _a,d,e,f_	(0.11)	4.62 _b,c,f,g_	(0.05)	4.31 _a,d,e,g_	(0.08)	9.15 ***
EC-ACT	4.85 _a_	(0.09)	4.52 _a,b_	(0.11)	4.12 _a,b,c_	(0.10)	4.94 _b,c,d_	(0.10)	5.00 _b,c,e_	(0.08)	4.70 _c,e,f_	(0.10)	4.98 _b,c,f,g_	(0.06)	4.73 _c,e,g_	(0.08)	9.68 ***
EC-ATT	3.79 _a_	(0.10)	3.57 _b_	(0.13)	3.49 _a,c_	(0.12)	4.35 _a,b,c,d_	(0.11)	4.52 _a,b,c,e_	(0.09)	4.09 _b,c,e,f_	(0.12)	4.58 _a,__b,c,f,g_	(0.06)	4.25 _a,b,c,e,g_	(0.09)	14.85 ***

Means with the same subscript are significantly different from each other: _a_ different from AN-R; _b_ different from AN-BP; _c_ different from BN; _d_ different from HC; _e_ different from Class II Obesity—BE; _f_ different from Class II Obesity + BE; _g_ different from Class III Obesity—BE. Abbreviations: BMI = body mass index; BIS = behavioral inhibition scale (punishment sensitivity); BAS = behavioral activation scale (reward sensitivity); TOT = total scale; DR = drive; FS = fun seeking; RR = reward sensitivity; EC = effortful control; INH = inhibitory control; ACT = activation control; ATT = attentional control. *** *p* < 0.001, ** *p* < 0.01.

## Data Availability

Data are available upon request at laurence.claes@kuleuven.be.
